# Facial erythema in frontal fibrosing alopecia: clinicopathological and dermoscopic features from a case series^؆^

**DOI:** 10.1016/j.abd.2026.501495

**Published:** 2026-11-07

**Authors:** Paula Gerlero, Marcello Menta Simonsen Nico, Neusa Yuriko Sakai Valente, Isabella Doche, Maria Cecilia Rivitti-Machado

**Affiliations:** Department of Dermatology, Hospital das Clínicas, Faculdade de Medicina, Universidade de São Paulo(HCFMUSP), São Paulo, SP, Brazil

Dear Editor,

Frontal Fibrosing Alopecia (FFA) is a primary lymphocytic scarring alopecia predominantly affecting the frontotemporal hairline and increasingly recognized as a global condition.^1^, ^2^ It is considered a variant of Lichen Planopilaris (LPP) due to histopathological similarities; however, FFA presents distinct clinical features and a broader spectrum of involvement, including extrafollicular manifestations.^3^, ^4^, ^5^ Facial papules and pigmentary changes have been described and are thought to be secondary to perifollicular inflammation and to the involvement of the dermoepidermal junction.^6^ Nevertheless, the significance and characterization of facial erythema in FFA remain poorly defined.

We aimed to describe the clinicopathological, dermoscopic, and ancillary features of facial erythema in patients with FFA. A retrospective comparative observational study including 14 patients with FFA-associated facial erythema and 11 gender- and age-matched controls with biopsy-proven rosacea, from Hospital das Clínicas, University of São Paulo, Brazil, between 2019 and 2025. Clinical, dermoscopic, histopathological, and laboratory data were collected. Skin biopsies (3-mm punch) from FFA patients with facial erythematous areas were analyzed using Hematoxylin-Eosin (H&E), Periodic Acid-Schiff (PAS), toluidine stain and Direct Immunofluorescence (DIF). The research was IRB-approved. Clinical characteristics of the case series were compared with those of a control group using the Fisher exact test for categorical variables and the Mann-Whitney *U*-test for continuous variables.

Patient characteristics are shown in Table 1. All FFA patients were women (phototypes III–V) with a mean age of 58.4 years (range 39–80). Most were postmenopausal (71.4%). All presented with typical frontotemporal alopecia, total or partial eyebrow alopecia, and variable loss of body hair. Facial erythema predominantly affected the cheeks and was occasionally associated with a burning sensation. Clinically, erythema alone was observed in 4-patients (28.6%), erythema with telangiectasias in 10 (71.4%) (Fig. 1a, Fig. 2a), and erythema with telangiectasias and pustules in 1 (7.1%). Dermoscopy revealed linear vessels arranged in a polygonal network in all but one patient (92.9%), (Fig. 1b, Fig. 2b). Additional findings included rosettes and follicular plugs (n = 4, 28.6%), and pustules with structureless orange areas (n = 1). Histopathological examination showed three main patterns: features compatible with Erythematotelangiectatic Rosacea (ETR) in 6 patients, characterized by superficial perivascular lymphohistiocytic infiltrate and vascular ectasia; LPP/FFA-like pattern in 3 patients, including lichenoid peri-infundibulitis and interface changes (Fig. 1c, Fig. 2c); and nonspecific findings in 5-patients, such as mild perivascular infiltrate, solar elastosis, and focal fibrosis. Demodex folliculorum was detected in 7- of 14-patients, across both rosacea and LPP/FFA-like histological patterns. DIF was negative for IgG, IgA, IgM, and C3 in 8- of 14-patients; in the remaining 6-patients, the DIF was nonspecific. Mast cell density ranged from 18- to 52-cells per high-power field (400×), with predominantly perivascular distribution, and was increased in both rosacea and LPP/FFA-like pattern cases, suggesting overlapping inflammatory pathways. Regarding treatment, patients received variable systemic and topical therapies, including hydroxychloroquine, 5-alpha reductase inhibitors, oral minoxidil, and topical agents. Notably, 7 patients showed improvement of facial erythema with topical nicotinamide 4% and photoprotection. Disease stabilization was achieved in 56.3% of cases, often requiring combination therapy.

**Table 1:** Clinical, dermoscopic, and histopathological characteristics of patients with FFA-associated facial erythema versus female rosacea controls.

Variable	FFA-associated facial erythema (n = 14)	Female rosacea controls (n = 11)	p-value
**Demographics**			
Median age, years (IQR)	57.0 (52.0–66.8)	52.0 (37.0–64.5)	0.380
Postmenopausal status	10 (71.4)	6 (54.5)	0.434
**Clinical features**			
Burning sensation	14 (100)	6 (54.5)	0.009
Telangiectasias	10 (71.4)	6 (54.5)	0.434
Papules/pustules	1 (7.1)	1 (9.1)	1.000
Eyebrow alopecia	14 (100)	0	<0.001
Body hair loss	9 (64.3)	0	0.001
**Dermoscopy**			
Polygonal vascular network	13 (92.9)	6 (54.5)	0.056
Rosettes	4 (28.6)	1 (9.1)	0.341
Follicular plugs	4 (28.6)	6 (54.5)	0.241
Structureless orange areas	1 (7.1)	2 (18.2)	0.565
**Histopathology**			
*Histology compatible with rosacea*	6 (42.9)	11 (100)	0.003
*Histology compatible with LPP/FFA*	3 (21.4)	0	0.230
Superficial perivascular infiltrate	11 (78.6)	11 (100)	0.230
Vascular ectasia/Telangiectasia	6 (42.9)	6 (54.5)	0.695
Perifolliculitis/Peri-infundibulitis	3 (21.4)	6 (54.5)	0.115
Interface dermatitis	3 (21.4)	1 (9.1)	0.604
Lichenoid changes	3 (21.4)	0	0.230
Follicular fibrosis	5 (35.7)	0	0.046
Solar elastosis	5 (35.7)	2 (18.2)	0.407
Demodex positivity	7 (50)	1 (9.1)	0.042

Values are n (%) unless otherwise indicated. Fisher exact test for categorical variables; Mann-Whitney *U*-test for age.

FFA, Frontal Fibrosing Alopecia; IQR, Interquartile Range; LPP, Lichen Planopilaris.

**Figure 1:**
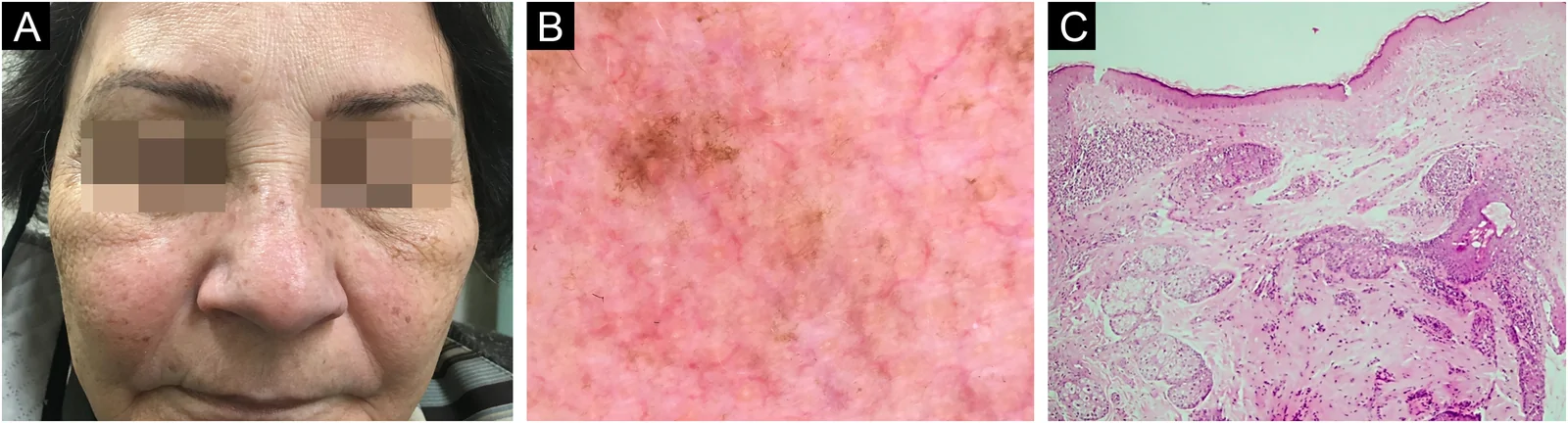
**Facial Lichen Planopilaris/Frontal Fibrosing Alopecia (LPP/FFA). Case 6** (a–c): (a) Clinical: facial erythema; (b) Dermoscopy: telangiectasia and short linear vessels in a polygonal network, (c) Histopathology: atrophic epidermis with a moderate perifollicular lymphohistiocytic infiltrate at the infundibulo-isthmic level (lichenoid peri-infundibulitis) (Hematoxylin & eosin,100×).

**Figure 2:**
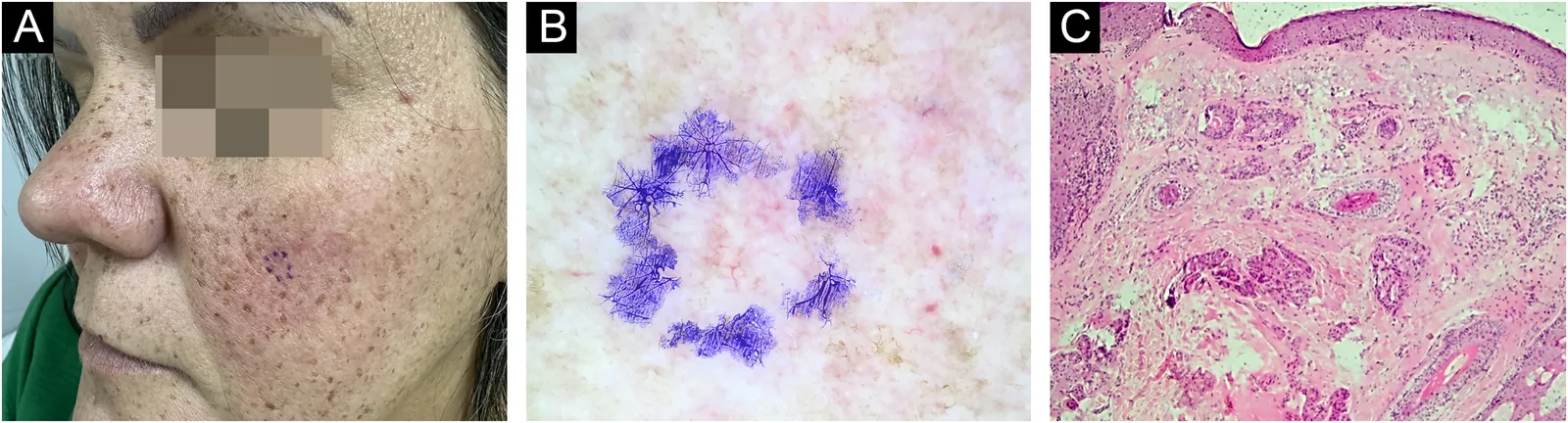
**Facial Lichen Planopilaris/Frontal Fibrosing Alopecia (LPP/FFA). Case 9** (a–c): (a) Clinical: facial erythema; (b) Dermoscopy: telangiectasia; The violet gentian marking on the cheek (b‒c) Indicates the planned site for a dermoscopy-guided 3-mm punch biopsy (c) Histopathology: atrophic epidermis with mild hyperkeratosis and a moderate perivascular and perifollicular lymphohistiocytic infiltrate in the dermis (Hematoxylin & eosin, 100×).

Compared with female rosacea controls, there was no significant difference between groups related to age and gender. As expected, eyebrow alopecia (100% vs. 0%, p < 0.001) and body hair loss (64.3% vs. 0%, p = 0.001) were significantly associated with FFA (Table 1). Histopathologically, follicular fibrosis was significantly more frequent in FFA-associated facial erythema (35.7% vs. 0%, p = 0.046), while lichenoid changes (21.4% vs. 0%, p = 0.230) and interface dermatitis (21.4% vs. 9.1%, p = 0.604) showed a similar but nonsignificant trend. Histology compatible with rosacea was significantly more frequent among controls (42.9% vs. 100%, p = 0.003), whereas superficial perivascular infiltrate (78.6% vs. 100%, p = 0.230) and perifolliculitis (21.4% vs. 54.5%, p = 0.115) did not differ significantly between groups. Demodex positivity was more common in patients with FFA-associated facial erythema than in rosacea controls (50% vs. 9.1%, p = 0.042).

The association between FFA and rosacea has been increasingly reported, with higher prevalence of rosacea in FFA patients compared to the general population.^5^, ^7^, ^8^ However, whether facial erythema in FFA may be an epiphenomenon or incidental factor or represents true rosacea, rosacea-like changes, or a distinct manifestation, remains unclear. Our findings support a heterogeneous spectrum. While a subset of patients exhibited clinicopathological features consistent with rosacea, others demonstrated interface dermatitis and lichenoid changes more suggestive of FFA involvement of facial skin. Shared pathogenic mechanisms may underlie this overlap. Both conditions involve dysregulation of innate immunity, vascular changes, and inflammatory mediators, including mast cells.^5^, ^9^ The increased mast cell density observed in our series, irrespective of histological pattern, further supports a common inflammatory pathway. Additionally, environmental factors, including contact allergens and UV exposure, as well as hormonal influences, may contribute to disease expression.^10^

The limitations of this study include its single-center design and relatively small sample size. In addition, not all clinical, dermoscopic, and histopathological data were available for the control group, precluding a comprehensive comparison of these parameters between cases and controls. Nevertheless, it highlights the heterogeneity of facial erythema in FFA and underscores the importance of clinicopathological correlation. Recognition of these patterns may have therapeutic implications, particularly in distinguishing patients who may benefit from rosacea-directed therapies, and also to consider FFA in every woman complaining of rosaceiform lesions.

In conclusion, facial erythema in FFA represents a spectrum ranging from rosacea-like features to lichenoid/interface dermatitis associated with FFA. Further studies are needed to clarify its pathogenesis and optimize management strategies.

## ORCID ID

Marcello Menta Simonsen Nico: 0000-0001-7968-0624

Neusa Yuriko Sakai Valente: 0000-0002-8065-2695

Isabella Doche: 0000-0003-1124-7175

Maria Cecilia Rivitti-Machado: 0000-0003-2910-7330

## Research data availability

The entire dataset supporting the results of this study was published in this article.

## Financial support

None declared.

## Authors’ contributions

Paula Gerlero: Study conception and design; data analysis and interpretation; critical review of important intellectual content; active participation in research supervision; intellectual participation in the therapeutic management of the case studies; final approval of the final version of the manuscript.

Neusa Yuriko Sakai Valente: Data analysis and interpretation; critical review of important intellectual content; final approval of the final version of the manuscript.

Isabella Doche: Active participation in research supervision; final approval of the final version of the manuscript.

Marcello Menta Simonsen Nico: Study conception and design; data analysis and interpretation; critical review of important intellectual content; active participation in research supervision; intellectual participation in the therapeutic management of the case studies; final approval of the final version of the manuscript.

Maria Cecilia Rivitti Machado: Study conception and design; data analysis and interpretation; critical review of important intellectual content; active participation in research supervision; intellectual participation in the therapeutic management of the case studies; final approval of the final version of the manuscript.

## Conflicts of interest

None declared.
